# Global Publishing Opportunities at the Journal of Aging & Social Policy

**DOI:** 10.1093/geroni/igaf122.1462

**Published:** 2025-12-31

**Authors:** Edward Miller, Elizabeth Simpson, Michael Gusmano, Pamela Nadash

**Affiliations:** University of Massachusetts Boston, Boston, Massachusetts, United States; University of Massachusetts Boston, Boston, Massachusetts, United States; Lehigh University, Bethlehem, Pennsylvania, United States; University of Massachusetts Boston, Boston, Massachusetts, United States

## Abstract

Policymakers, practitioners, and researchers need a balanced, thoughtful, and analytical resource to meet the challenge of global aging at a rate that’s historically unprecedented. The Journal of Aging & Social Policy (JASP), which was founded in 1989, serves this role by drawing contributions from an international panel of policy analysts and scholars who assume an interdisciplinary perspective in examining and analyzing critical phenomena that affect aging and the development and implementation of programs for older adults from a global perspective. Study settings extend beyond the United States to include Europe, the Middle East, Australia, Latin America, Asia, and the Asia-Pacific rim. This presentation will document the scope, content, and focus of JASP, including the rise of international submissions, which now account for approximately half of articles published. Opportunities for publishing in JASP will be discussed; so too will strategies for navigating the peer-review process successfully.

